# A Cognitive-Behavioral Model of Apathy in Parkinson's Disease

**DOI:** 10.1155/2024/2820257

**Published:** 2024-08-31

**Authors:** Olivia Plant, Annika Kienast, Daniel S. Drew, Elitsa D. Slavkova, Kinan Muhammed, Helen Kennerley, Masud Husain

**Affiliations:** ^1^ Department of Experimental Psychology University of Oxford, Oxford, UK; ^2^ Oxford Cognitive Therapy Centre Oxford Health NHS Foundation Trust, Oxford, UK; ^3^ Nuffield Department of Clinical Neurosciences University of Oxford, Oxford, UK; ^4^ Department of Neurology John Radcliffe Hospital Oxford University Hospitals NHS Foundation Trust, Oxford, UK; ^5^ Wellcome Centre for Integrative Neuroimaging University of Oxford, Oxford, UK

## Abstract

Apathy is recognized to be a common, disabling syndrome that occurs across a range of psychiatric and neurological conditions, including Parkinson's disease. It can have a significant impact on quality of life, both for people affected and those around them. Currently, there are no established, evidence-based treatments for this debilitating syndrome. Assessment and treatment have been complicated by overlaps with depression and anhedonia, as well as a lack of understanding of the underlying mechanisms. Emerging lines of evidence conceptualize apathy as a reduction of motivation associated with disordered effort-based decision-making and dysfunction of distinct neural circuitry between the basal ganglia and medial prefrontal cortex. Here, we introduce a novel cognitive-behavioral framework that can inform a clinician's conceptualization and treatment of apathy, using cognitive-behavioral therapy (CBT) techniques. We focus on people with Parkinson's disease in our model, but our approach is transdiagnostic and can be applied to other conditions. It considers both individual targets for therapy as well as maintenance and intervention at a systemic level. The generalizability and parsimony of the framework provides a structured assessment and formulation of apathy, while also allowing clinicians to remain sensitive to other neuropsychiatric symptoms that can occur alongside apathy, such as depression and anxiety.

## 1. Introduction

Apathy is a syndrome characterized by lack of motivation leading to a reduction in goal-directed behavior [1]. It occurs across a range of neurodegenerative, inflammatory, infectious, and traumatic brain pathologies in addition to being a symptom of psychiatric conditions including schizophrenia and major depressive disorder [2]. People with apathy often report that they “cannot be bothered,” that “lots of activities don't seem worth the effort,” or they have diminished “get up and go.” Understandably, this has led to difficulties in separating apathy from depression and anhedonia, but studies have now shown that although some people can suffer from both apathy and depression, or both apathy and anhedonia, there are individuals who suffer from apathy alone and are neither depressed nor anhedonic [3, 4].

There has been a growing interest in apathy, its underlying mechanisms, and appropriate treatment targets, particularly because its presence independently predicts poorer quality of life, as well as worse functional prognosis and caregiver burden [5, 6]. While there have been trials investigating pharmacological and behavioral interventions [7–10], there are no established treatments.

One important condition in which apathy has been studied in detail is Parkinson's disease (PD) [4]. Although there are some pharmacological studies on the treatment of apathy in PD [11, 12], none have led to formal recommendations for the management of patients with the condition. There is growing evidence that cognitive-behavioral therapy (CBT) can be an effective treatment for several neuropsychiatric features in PD including depression and insomnia [13–15]. Here, we present a novel framework that captures a cognitive-behavioral and systemic understanding of apathy, based on our clinical experience with sufferers of this syndrome. Although our focus is on PD, the framework provides a testable conceptualization of apathy more generally and presents clear options for therapeutic targets that might be applied to apathy in the context of other neurological and psychiatric conditions.

## 2. Phenomenology

Apathy is a complex phenomenon with several potential underlying mechanisms [4, 16, 17]. These range from impairments in the ability to generate options for behavior (option generation); cost-benefit decision-making prior to committing to a behavioral activity; persisting with a behavior; and being able to evaluate and learn the costs and benefits of making a particular choice of behavior [18]. It has been proposed that apathy results from disruption of distinct neural circuitry linking the basal ganglia and prefrontal cortex [17, 19].

A neurocognitive framework of apathy across different disorders [19] postulates that an interconnected group of brain regions, with the anterior cingulate cortex (ACC) and ventral striatum (VS) at its core, plays a crucial role in normal motivated behaviour. A key function of the ACC and the VS is thought to be the representation of the value of potential actions, which is central to motivated, goal-directed behaviour. The results of neuroimaging studies across several different diseases have shown that these functions are disrupted in apathetic patients. In PD specifically, regions in the medial and lateral prefrontal cortex and the midbrain ventral tegmental area (VTA) are additionally thought to be linked to the development of apathy [19]. Animal studies have shown that the VTA is a key source of dopaminergic projections to the nucleus accumbens (NAc, the equivalent of the VS in humans) and to the prefrontal cortex [20], and dopamine has been linked with motivated behavior and the processing of rewards [21, 22]. Dopaminergic dysfunction in PD is considered to be an important contributor to apathy in PD [19]. Apathy is therefore considered to be a manifestation of the neurodegenerative processes in PD rather than a psychological response to the disease [23].

Idiopathic PD is a common neurodegenerative disorder associated with a dopaminergic deficit [24]. Although it is characterized by motor symptoms, PD has also been described as the “quintessential neuropsychiatric disorder” [25], with anxiety, apathy, depression, psychosis and impulse control and sleep disorders as being possible symptoms [26]. Neuropsychiatric features can often precede motor symptoms by years or even decades [27]. The pathophysiology of these symptoms is likely complex and not solely attributable to dopaminergic neurotransmitter systems. It is likely that serotonergic, cholinergic, and noradrenergic systems are also implicated [28]. Neuropsychiatric symptoms in PD have a significant impact on quality of life, lead to increased caregiver distress [29], reduce cognitive status [30], and are therefore of diagnostic and therapeutic significance [31].

The prevalence of apathy in PD patients ranges from 17 to 70%, depending on the assessment scales used and populations studied [23, 29, 32], and has been shown to occur at all stages of the disease. Apathy has also been conceptualized as a multidimensional construct with dissociable domains. While there are several frameworks of apathy postulating different domains, there seems to be a consensus on the presence of a behavioral/cognitive and an emotional axis of apathy [17, 33–36]. However, the general model for treating apathy proposed here operates without the need to formally delineate subtypes of apathy.

Apathy and depression share several features and are thus often conflated. Assessment and treatment of apathy therefore remains challenging and complex. A general reduction in activity is a common symptom, maintaining factor of depression, and is often driven by reduced motivation or apathy towards certain activities. As a result, this can make it difficult to distinguish between the two syndromes in clinical populations and we therefore considered it to be important to highlight these differences so that clinicians are able to understand where there are possible overlaps [37], and also to differentiate between them in order to understand the reasons for people's inactivity and develop tailored personalized treatments. The motivational loss associated with apathy is not solely attributable to emotional distress [38], such as that seen in depression. Apathy and depression seem dissociable but can nevertheless occur together in PD [23, 29, 39]. Loss of motivation can indeed be a key diagnostic feature of depression as included in the DSM-V [40]. There are however symptoms unique to depression such as suicidality, despair, and loss of appetite that are not observed in apathy alone. It has therefore been thought that some of the differences between depression and apathy in PD occur because of differing underlying neurobiological mechanisms [41, 42]. A recent review concerning these syndromes considers in-depth features that might be shared and common to them both, and those that might differentiate them [37], although it is clear that different patients may suffer from different constellations of underlying symptoms.

Like apathy, anhedonia has generated much attention as a neuropsychiatric symptom in PD. This phenomenon has considerable overlaps with apathy in different disorders [43]. Anhedonia is defined as a state in which an individual cannot derive pleasure from behaviors and interactions that they were once able to. More recently, this definition has been updated to reflect a loss of interest or motivation to seek out pleasure in the first place [44]. Anhedonia has been divided into a consummatory or “liking” component (the satisfaction derived from consuming a reward) and an anticipatory or “wanting” component (the desire to obtain a specific reward) [45]. These components likely originate from separate neural mechanisms [44], with some researchers suggesting that apathy is more closely linked to “wanting,” and depression is more associated with “liking” [46, 47]. Across neurodegenerative disorders, most research that has been conducted on the relationship between apathy and anhedonia has been in PD, but even here this has been at the level of descriptive, questionnaire studies, so mechanistic differences have not been clearly elucidated [48].

Despite potential commonalities across apathy, depression, and anhedonia, it remains unclear exactly where these lie [18]. Our intention is that the following model will allow clinicians to understand, conceptualize, and treat apathy in PD, while also remaining sensitive to other syndromes that might occur alongside it. But, for the sake of simplicity and given the current levels of understanding, we have not added anhedonia specifically into the current model.

## 3. A Cognitive-Behavioral Model of Apathy

CBT is a versatile therapy that is widely applied to help people suffering from a range of emotional conditions. It has been highly successful in treating depression and anxiety in people without any other brain disease, as well as in patients with neurodegenerative conditions, including PD [49, 50], in which there is evidence that it can modulate brain circuits, for example, when CBT is applied for anxiety [51]. Thus, although the targets of CBT are the psychological processes that are manifestations of altered neural function, across different brain conditions, it has the potential to modulate neural circuitry and thereby help change people's behavioural and cognitive habits.

To the best of our knowledge, there is no existing cognitive-behavioral conceptualization of apathy, like there is for those with PD experiencing anxiety and depression [52]. The present novel framework captures a cognitive-behavioral and systemic understanding across a range of difficulties faced by people with PD who suffer from apathy.

### 3.1. Model Development

**Figure 1:**
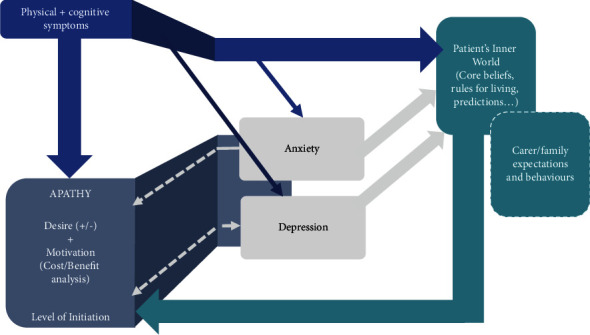
A CBT model of apathy. The first section of the model allows patients to understand their experience of apathy and PD, and why specifically they are affected. We conceptualize apathy as the will (or lack thereof) to complete a behavior. This is influenced by an individual's level of motivation, which is in part influenced by a process of disordered effort-based decision-making. Together, these factors influence whether one initiates a behavior. Due to the known impact of long-term conditions on mental health [53], the impact of physical and cognitive symptoms, such as tremor, rigidity, or memory problems, on one's decision-making process is also considered, as well as how they might influence often observed comorbidities such as anxiety and depression. These symptoms in turn affect how apathy is experienced. In addition, we emphasize how physical symptoms may interact with the patient's core beliefs and underlying assumptions which, as well as their family's idiosyncratic experiences and beliefs, directly influence the experience of apathy.

Commonalities in the maintenance processes underpinning apathy were conceptualized into a model (Figure 1), with the aim of being parsimonious, so that the framework would be accessible to patients. The model draws on clinical observations, interviews, and assessment in addition to the neuroscientific literature on apathy.

Fourteen patients were assessed and recruited through the Oxford Cognitive Disorders Clinic in the neurology department. They were all assessed with clinical interviews of themselves and caregivers, and physical examination by qualified neurologists. Cognitive assessment using the Addenbrookes Cognitive Examination-III was performed by psychologists, together with screening questionnaire assessments of depression, apathy, and anhedonia. Apathy was measured using a standardized clinical measure, the Lille Apathy Rating Scale (LARS), which has been validated in PD [34]. Depression was assessed via the Beck Depression Inventory (BDI-II), and anhedonia was assessed via the Snaith-Hamilton-Pleasure-scale (SHAPS). All of the PD patients had clinically significant apathy (LARS-self report or LARS-caregiver report score > −21). One also suffered from moderate depression (BDI-II of 20–28), two had slight depression (BDI-II of 14–19), five had minimal depression (BDI-II of 9–13), while the other six patients had pure apathy. Patients with higher depression also showed more anhedonia (higher SHAPS score). However, the majority were not anhedonic (10 patients had a SHAPS score of 0).

Patients and their caregivers were then interviewed at the Department of Experimental Psychology by a team of four psychologists under the supervision of a neurological (MH) and a CBT supervisor (HK). At the start of the session, we spoke to patients and their partner/caregiver together. Consent forms were completed, and the purpose and process of the interview was explained to both individuals. Patients were then assessed for 2 hours, and their partners/caregivers were also interviewed simultaneously in a separate room for an hour by a member of our team. The patients' experiences were formulated by asking them about their individual experience of apathy: their cognitions, emotions, physical/bodily sensations, and behaviors in situations where problems with motivation occurred. They were additionally asked about triggers and modifiers, commonalities and exceptions, consequences (e.g., family reactions) and coping strategies. From this information, an individualized formulation of apathy was drawn up for each participant and reviewed with them to confirm that it fitted with their experience. Each patient brought along with them to the interview sessions someone who knew them well, in most cases their partner. We interviewed these individuals on *their* perception of the patient's apathy and asked them to complete the informant version of the Lille Apathy Rating Scale (LARS-caregiver), which has similarly been validated for the caregivers of people with PD [54].

The experiences of some of the patients could be shared simply using a generic cognitive-behavioral Beckian formulation [55]. However, for many problems that we formulated, this framework did not capture all relevant facets. The Beckian model primarily relates to emotional disorders and does not specifically consider the role of long-term health conditions, such as PD, or indeed other brain disorders (e.g. Alzheimer's disease or vascular dementia) in which apathy can occur. While the model is very flexible and could be adapted to capture apathy and the relationship it has with PD, this would require a highly skilled clinician to be able to capture the nuanced links between these features, and any other possible comorbidities. Our model simplifies this process for clinicians and, importantly, makes it more accessible to patients and their families. We therefore developed a novel heuristic based on Beck's original framework. To develop this current model (Figure 1), the team repeatedly reviewed the formulations of all patients, to identify commonalities and patterns across cases, refining the model throughout the process.

During the process of clinical supervision with HK, authors OP, AK, DD, and ES developed four iterations of the model which were adjusted based on client interviews and feedback. Each iteration of the framework was piloted with the patients to gain feedback concerning its strengths and weaknesses and to identify common patterns across cases. The purpose of this refinement through piloting was not only to collect more detail but also to assess the model's “user-friendliness.” The iteration we present in our paper is the most developed version of the model.

### 3.2. Final Model

In constructing a new cognitive-behavioral understanding of apathy in PD, we considered the following aspects of patients' experiences:

- The problem of apathy and how it might be conceptualized
- The impact of patients' symptoms on motivation
- The “inner world” of *both* the patient and the family/caregiver
- The effects of comorbidities, including the influence of depression and/or anxiety
- The response of family/caregiver to the patient

#### 3.2.1. The Problem of Apathy

We conceptualized apathy as a person's will (or lack thereof) to do something. This is governed by their level of motivation, influenced, in part, by a process of effort-based decision-making [18]. Specifically, if a person perceives the cost of carrying out an action is greater than the benefits, it will influence their motivation. The combination of desire and motivation will determine whether an action is initiated or not. Examples below illustrate these principles:

*“Patient A described their experience of apathy as a feeling of, “I just can't be bothered.” They said, “I know I should be doing it, but I tell myself it can wait.” The effort, or cost, of doing the chore would seem very high, while the benefit, in their perception, would be negligible. They would feel desire to do more pleasurable activities, like going for a walk. However, the effort of putting on their shoes and jacket, and the anticipated exhaustion afterwards was perceived as so high that there would seem little point in initiating the action. There were other, previously enjoyed activities like gardening, for which they had lost desire.”*

*Patient B used to love meeting people and to put effort into their appearance. Apathy made them decline invitations to social gatherings or make up excuses so they wouldn't have to go. The effort of getting ready seemed insurmountable and they imagined the social interactions would be exhausting. They said, “While I used to be a very active person–never sitting down, always busy–I am now quite content to just sit on the couch for hours, doing nothing, or watching the birds in the garden.”*

#### 3.2.2. Physical and Cognitive Symptoms

Salkovskis and colleagues [56] have argued that physical symptoms should be incorporated into cognitive-behavioral formulations as they are an important dimension in the challenges faced by people with a preexisting illness. The influences of physical symptoms on apathy were incorporated into our model. Symptoms include: tremor, slowness of movement, rigidity, dyskinesia, freezing, hypomimia, drooling, shuffling, and poor voice projection. Cognitive deficits also occur in PD, and for the purposes of parsimony, we also include these under physical symptoms. Impairments in executive function, a common cognitive feature in PD, can also determine an individual's willingness to engage. The extent to which physical symptoms occur in patients varies, but they have an impact on motivation.

Where motor and cognitive symptoms are deemed to influence a patient's decision to engage in behaviors, we recommend carefully assessing current symptoms and the ways in which these impede activity levels of a patient, both from a functional and psychological perspective. Below are some illustrative examples:

*“Patient C used to be a good public speaker. With the onset of PD, their voice got softer and no longer carried across the room. Additionally, as a result of PD related cognitive changes, they experienced word finding difficulties. While previously they were actively involved in their community, participating, and organizing meetings and discussions, they now felt incapable of doing so with their articulation and voice affected by PD.”*

*“Patient D used to love going to the cinema with a friend. After being diagnosed with Parkinson's, they made excuses whenever they were invited to see a movie. They were worried that their tremor during the film would shake the entire row of seats, and everyone would stare at them.”*

*“Patient B frequently froze while walking and the time windows in which their medications reduced the likelihood of this happening were narrowing. Consequently, walking took much longer, and it no longer seemed worth the effort to undertake what had once been their daily routine to go to buy the newspaper.”*

#### 3.2.3. The Patient and Family's Inner World

Our model considers *both* the patient and their family's or caregiver's idiosyncratic beliefs. Incorporating systemic cognitive factors into the model allows for the relevant thoughts and assumptions of patients and their carers to be understood and addressed when necessary. For example:

*“Patient D held very high expectations for themselves. One of their rules was, “I should be able to do things as I did before Parkinson's. Otherwise, there is no point in trying.” They had once loved helping their family with any handiwork that needed completing. Due to PD symptoms, they now struggled to complete these tasks with the same precision and ease as before, consequently perceiving that instead of being a help, they would create more problems and felt it better not to try.”*

*“Patient D's family members were all very active and busy. They shared the belief that, “as long as you can physically do it, there is no reason not to.” From their perspective, it was all a question of “mind-over-matter.” Because Patient D's physical symptoms varied a lot, and sometimes they would be as capable as they were before PD, the family underestimated the severity of the patient's physical impairments and expected them to overcome their symptoms and complete tasks to the same standard as before.”*

#### 3.2.4. Comorbidity and the Influence of Depression and/or Anxiety

Motivational deficits form part of the diagnostic criteria for depressive disorders [40]. As previously stated, although there can be overlap between apathy and depression, each syndrome can occur in isolation [39]. In our patient population, we observed both apathy and depression as dissociable phenomena. Nevertheless, because they can be experienced concurrently and due to the high incidence of depression in PD [29], we also assessed patients for depression and, if present, considered how it might influence apathy.

As with any long-term condition, it is crucial to assess the extent to which individuals perceive themselves as disabled, having a poor quality of life and their views on their prognosis. All are factors that can influence mood, as well as motivational state.

*“Patient A suffered from depression alongside apathy. Perfectionism and very high expectations of themselves led to disappointment, low self-esteem, and low mood. They then set unrealistic compensatory high standards, comparing their abilities to their younger and healthier self, and criticizing themselves as a “couch-potato”.*

If a patient also suffers from anxiety, particularly health-related anxiety, and this causes excessive worry about engaging in certain activities, then avoidance or the implementation of safety behaviors might be behavioral strategies used to minimize the threat that Parkinson's poses to their ability to carry out a task. In the context of a neurodegenerative condition, it is important also to assess if there are fears concerning the future trajectory of disease progression. These may influence how a patient assesses whether a particular rewarding outcome is worth the effort needed to complete a task.

*“After being diagnosed with PD, Patient B worried about suffering from increasing symptoms as the disease progressed. They were specifically anxious about becoming incontinent and would therefore not leave the house for long periods or go to places unless they knew the whereabouts of the nearest toilet, and they would always carry a change of clothes with them. This reduced their options of places to visit and increased the effort of going out.”*

#### 3.2.5. Unhelpful Behavioural Strategies and Cognitive Processing Styles

Through interviews, we have identified four different cognitive and behavioral responses to apathy, reflecting the patients', families', and/or caregivers' responses.

**Figure 2:**
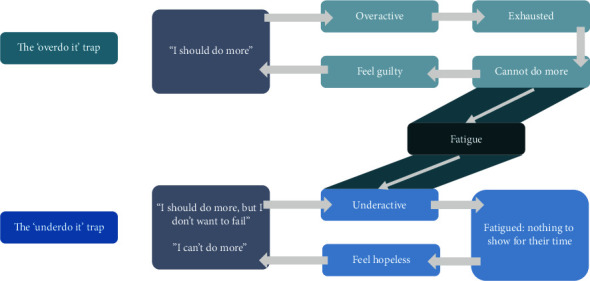
Maintenance cycles of apathy: What the person with apathy does. This part of the model displays the two “maintaining” behavioral cycles in which the patient can get caught (“traps”). They capture and explain why the problem of apathy persists and can be used to guide treatment. The “overdo it” trap is driven by unrealistic high standards or guilt cognitions, leading the patients to being overactive and thereby exhausting themselves. This causes lack of investment in further effort, making them feel guilty and therefore reinforcing guilt cognitions. The “underdo it” trap begins with negative automatic thoughts regarding patients' own abilities, which deters them from engaging in activity. Underactivity leads to a sensation of fatigue, not having anything to show any useful result or outcome and consequently to a feeling of hopelessness. This, in turn, feeds back into the negative automatic thoughts regarding a patient's own ability. The “overdo it” trap can turn into the “underdo it” trap: if overactivity leads to fatigue and therefore to not initiating activities, thus being underactive.

*(1) What the Person with Apathy Does*. The person with apathy can fall into two different behavior styles, each potentially detrimental (Figure 2). Hence, we refer to these as “traps.” First, a person may have unrealistic high standards or guilt that drives them towards overactivity–the “overdo it” trap. The result of this overactive behavior is patient exhaustion, discomfort, and fatigue, which in turn leads to beliefs that they cannot do more and further guilt or self-criticism. Such appraisals produce negative emotional consequences such as low mood and further low motivation. These responses negatively reinforce unhelpful behavioral strategies including stopping certain activities or rejecting them altogether as opposed to modifying them in line with what they might be capable of.

*“Patient A enjoyed going for walks, so they would go for a long walk but become exhausted. After returning home, they would feel very tired and take a nap. They would awake refreshed, but also guilty about resting and not doing something more useful. They would think, “I am a couch potato, I used to be able to go for much longer walks without resting,” which in turn would lower their mood and worsen apathy, affecting their evaluation of the costs and benefits of future walks.”*

In contrast, the “underdo it” trap is characterized by beliefs such as “I should do more, but I do not want to fail” or “I can't do more.” The result is that the individual with apathy is underactive and can paradoxically develop a sensation of feeling further inert and fatigued due to inactivity and have nothing to show for their time. This can feed feelings of hopelessness and loss of confidence which, in turn, can promote negative cognitions, reducing mood and motivation further.

Patients can fall into just one of the traps, but, commonly, they fall into both, depending on the situation. In addition, the sensation of fatigue forms a link between the two cycles. When patients are exhausted from overdoing it and feel fatigued, they can believe they are not able to do anything or fear failing. This can result in underactivity, stopping, or not initiating certain activities instead of modifying them according to what they might be capable of.

*“Patient C lost their motivation to engage in their usual hobby of sewing. They were unable to sew as well as previously, becoming frustrated and exhausted with their efforts which they perceived as a failure. This created a feeling of fatigue and hopelessness that discouraged them from engaging with an activity that once brought great pleasure.”*

**Figure 3:**
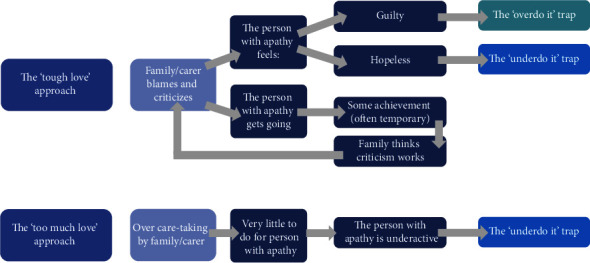
Systemic maintenance cycles: What the carer/family does. This part of the model displays the two “maintaining” cycles in which the person with apathy's close social system can get caught (“traps”). In the “tough love” approach, the family criticizes the patient for being inactive. This can make patients feel either guilty, leading them to fall into the “overdo it” trap, or hopeless, leading to the “underdo it” trap. Blame and criticism can cause the patients to get going and potentially to temporary achievement. This can in turn reinforce the family's response. The “too much love” approach means that the family is overprotective and does everything for the patients, leaving very little for them to do. This can reduce patient activity and lead them to the “underdo it” trap.

*(2) What the Family Does*. We have observed that the role of the family and/or caregiver is of utmost importance, as these interactions can influence the cycles detailed above. Patients' close social contacts can behave in ways that can unintentionally exacerbate their problem of apathy (systemic traps, Figure 3).

When the family/carer is distressed by a patient's lack of concern or indifference about activity levels or the activity levels themselves, the patient might be criticized by those close to them. We refer to this as the “Tough Love” approach. Such responses can feed feelings of guilt and drive a patient's unrelenting high standards (the “overdo it” trap), leading to unsustainable short-term behavioral change. This can prompt feelings of hopelessness, which activate the “underdo it” trap.

*“Patient A's spouse would say, “You were going to take out the rubbish. You forgot again.” This would lead to A's automatic thought, “No! I don't want to do this anyway.” The feeling of defiance was followed by guilt, shame, and worthlessness, so they would complete the chore, causing their spouse to believe that criticism and blame were effective motivators.”*

On the other hand, the “Too Much Love” approach is borne out of an overprotective caregiving style. This can involve carers doing things for the patient when it is possible for the patient to do this themselves, perhaps with more encouragement or support. This strategy can have deleterious effects on the patient's levels of self-generated action, thus undermining progress.

*“Patient C's spouse would not want to impose on their partner and instead complete all the household chores and cleaning themself. This would leave little for the patient to do, resulting in a lack of purpose, further undermining their need to initiate activities at home.”*

Based on the apparent important influence of family members/caregivers in motivating and/or demotivating patients, a systemic CBT approach might be a more effective treatment path than one solely focused on the patient's cognitive and behavioral responses. However, only the elements of the model that are relevant for a patient would be shared with that person. Thus, if there were no systemic traps, then the shared formulation would not include those cycles.

In summary, our novel and parsimonious model is an adaptation of Beck's original cognitive-behavioral heuristic [57]. It offers clinicians a framework for understanding, conceptualizing, and treating behavioral apathy in patients with PD. Due to its transdiagnostic nature, it can be applied to other conditions in which apathy presents. It offers both individual targets for therapy as well as maintenance and intervention opportunities at a systemic level. The model also allows the possibility of incorporating longitudinal factors that can allow a patient to consider “why me,” contextualizing their difficulties, thus offering the possibility of shifting self-blame or bewilderment about their struggles [58]. The clinician can then tease out core beliefs and underlying assumptions later in therapy if relevant to the patient.

## 4. Patient Feedback

The model was presented at a PD conference, “Fighting Fit” [59]. Attendees included patients with PD and their partners/caregivers. They were given a leaflet, designed by our team, focusing on apathy and fatigue in PD with an emphasis on the maintenance factors perpetuating apathy (“traps”). Following the event, we received positive feedback on this aspect of the model. This feedback was in the form of patient and patient family comments which were transcribed and sent via e-mail. Patients noted that it was helpful to understand that apathy is not merely laziness but attributable to biological changes that occur as a result of PD.

In particular, patients and family members/caregivers also found it helpful to understand how these symptoms can be perpetuated through cognitive-behavioral “traps.” This understanding instilled hope that apathy could be alleviated by breaking recognized patterns, but attendees felt that they needed further guidance to overcome these. A course of CBT for apathy, facilitated by a specialist, could be implemented to train patients and family/caregivers to identify and tackle unhelpful cognitive-behavioral patterns independently. To this end, education of these factors could be very beneficial when working therapeutically with patients with apathy and their families.

## 5. Therapeutic Interventions

Therapeutic interventions derived from the proposed model would be based on the identified maintenance cycles: the “overdo it,” “underdo it,” and “systemic” “traps.” The goal would be to achieve a balance between activity and rest for the patient as well as between support and independence from the family. Psychoeducation would play an important role in improving patients' and carers' understanding of apathy, highlighting how it is a symptom of PD and has a neurological underpinning and is not mere laziness. Furthermore, identification of the cognitive processes contributing to apathy, such as ability to generate options for behavior, make effort-based decisions in relation to behavior, and persist and/or learn from a behavior may aid the selection of appropriate interventions and approaches in CBT.

Therapy would focus on teaching patients proven and well-tested CBT skills to accomplish this balance. This may include standard behavioral techniques, such as activity monitoring and scheduling (with current levels of activity ranked for pleasure and achievement), and goal attainment supported through scheduling, graded practice and review [57]. This would be paced in keeping with a patient's abilities and might need to be informed by other medical professionals. Another (simple) behavioral technique is applied relaxation, which could help patients or caretakers whose stress makes them vulnerable to unhelpful cognitions and behaviors.

Cognitive methods might also be employed. These could include “standard” Beckian techniques, such as: identification and modification of unrealistic automatic thoughts; unhelpful assumptions or cognitive biases concerning self or activities; reassessment of standards, life goals, and values; distraction; and problem-solving. Again, intervention would be tailored to reflect a patient's (or family member's) needs and abilities.

Prospective mental imagery, the capacity to preexperience future events based on preexisting memory, has been shown to be effective in motivating depressed clients [60]. Through this technique, individuals have been shown to emulate real-life experiences that can impact emotion and behavior [60–62]. In patients struggling with apathy, this cognitive method could allow the individual to picture how they might approach and complete a task. In addition, it could strengthen their capacity to imagine the (beneficial) outcome of their actions, thereby affecting their cost-benefit evaluation of initiating or sustaining a behavior. In this way, prospective mental imagery development could offer a potential treatment for those with apathy.

Following psychoeducation of the family about common traps and the underlying systemic processes reinforcing apathy, we recommend identifying family cognitions and behaviors that might fuel a systemic trap. An exploration of alternative perspectives and teaching alternative behaviors for supporting and promoting independence in those suffering from apathy could then follow.

A cognitive-behavioral conceptualization captures habitual problem cognitions and behaviors, thus facilitating anticipation of problems and obstacles in treatment. This means that intervention can embrace effective problem-solving and relapse management so that the patients and caregivers develop long-term coping skills.

Overall, based on this model, therapy for apathy would not require the development of new therapeutic methods but use established and proven CBT techniques guided by the patient's apathy-focused formulation. Given our experience thus far, we propose that therapy for apathy in PD could potentially be conducted in twelve consecutive sessions with a possibility to extend to twenty sessions if necessary.

## 6. Conclusion

We have outlined a novel, parsimonious, and transdiagnostic framework that can be used to deliver a personalized psychological intervention for apathy in PD. The proposed model is consistent with the main clinical features of apathy, while also capturing the cognitive-behavioral nuances of this transdiagnostic phenomenon. It illustrates maintenance factors that likely exacerbate and cause the apathy presentation to persist, the influence of disease-specific symptoms on an individual's effort-based decision-making processes, and the role of the wider system in the understanding and treatment of this condition. The proposed model provides a compassionate and systemic CBT framework for treatment that seems to be user-friendly. The generalizability and parsimony of this framework also allows clinicians to remain sensitive to comorbidities such as depression and forms of anxiety that can occur alongside apathy. These can influence the experience of apathy and might also warrant therapeutic intervention. It is hoped that future research will further investigate this model and its implications for the treatment of apathy in PD, as well as apathy associated with other brain disorders.

## Data Availability

The questionnaire data used to support the findings of this study are available from the corresponding author upon request. The individual clinical formulations and interview data are however not available due to ethical reasons.
